# Too little but not too late: Results of a literature review to improve routine immunization programs in developing countries

**DOI:** 10.1186/1472-6963-8-134

**Published:** 2008-06-21

**Authors:** Tove K Ryman, Vance Dietz, K Lisa Cairns

**Affiliations:** 1 Global Immunization Division, Centers for Disease Control and Prevention, 1600 Clifton Road MS-E05, Atlanta GA 30333, USA

## Abstract

**Background:**

Globally, immunization services have been the center of renewed interest with increased funding to improve services, acceleration of the introduction of new vaccines, and the development of a health systems approach to improve vaccine delivery. Much of the credit for the increased attention is due to the work of the GAVI Alliance and to new funding streams. If routine immunization programs are to take full advantage of the newly available resources, managers need to understand the range of proven strategies and approaches to deliver vaccines to reduce the incidence of diseases. In this paper, we present strategies that may be used at the sub-national level to improve routine immunization programs.

**Methods:**

We conducted a systematic review of studies and projects reported in the published and gray literature. Each paper that met our inclusion criteria was rated based on methodological rigor and data were systematically abstracted. Routine-immunization – specific papers with a methodological rigor rating of greater than 60% and with conclusive results were reported.

**Results:**

Greater than 11,000 papers were identified, of which 60 met our inclusion criteria and 25 papers were reported. Papers were grouped into four strategy approaches: bringing immunizations closer to communities (n = 11), using information dissemination to increase demand for vaccination (n = 3), changing practices in fixed sites (n = 4), and using innovative management practices (n = 7).

**Conclusion:**

Immunization programs are at a historical crossroads in terms of developing new funding streams, introducing new vaccines, and responding to the global interest in the health systems approach to improving immunization delivery. However, to complement this, actual service delivery needs to be strengthened and program managers must be aware of proven strategies. Much was learned from the 25 papers, such as the use of non-health workers to provide numerous services at the community level. However it was startling to see how few papers were identified and in particular how few were of strong scientific quality. Further well-designed and well-conducted scientific research is warranted. Proposed areas of additional research include integration of additional services with immunization delivery, collaboration of immunization programs with new partners, best approaches to new vaccine introduction, and how to improve service delivery.

## Background

Immunizations are a cornerstone of public health: the World Health Organization (WHO) estimates, that in 2006, immunizations saved two to three million lives [1]. Nonetheless, in that same year 1.4 million children are estimated to have died from vaccine preventable diseases (measles, *Haemophilus influenzae *type B [Hib], pertussis, tetanus, yellow fever, and poliomyelitis), a reflection of the incomplete coverage with existing vaccines that persists in many parts of the world [1]. In 2006, of the 157 WHO member states defined as "developing", only 42 (27%) had three doses of diptheria-pertussis-tetanus (DPT) coverage greater than 80% in all districts [2]. At the same time, new opportunities exist to strengthen immunization coverage in developing countries, such as increased funding through platforms such as the Global Immunization Vision and Strategy (GIVS), as well as novel ideas for integration with other health services. These recent developments have encouraged a macro-analytic approach to ensure that systems function so that children receive needed vaccines. While these new approaches are welcomed, at the micro level, immunization service delivery in health facilities needs to be strengthened.

Immunization programs need continued support with proven strategies and fresh approaches to reduce the incidence of diseases that may be prevented through the use of traditional vaccines, and to permit the effective introduction of new vaccines. There are 23 new or improved vaccines for children and adolescents in development [3]. Integrating these vaccines into routine programs will substantially increase the needed expenditure on routine immunizations. To fully take advantage of these new vaccines it is essential to identify and utilize proven strategies for improving routine immunization programs at the service delivery level. Despite the attention that global immunization has attracted in recent years in terms of the introduction of new vaccines and the strengthening of health systems, there is a clear need to ensure that program managers are aware of what strategies at the health facility level will be needed to strengthen programs. To help identify these strategies a review of gray literature and a systematic review of published literature were conducted. In this paper, we present the strategies that may be used at the community or facility level that have been shown to strengthen routine immunization programs. This review builds on two similar reviews of immunization service strategies in developing countries which were published between 2004 and 2005, one of published literature and the other of gray literature [4,5]. Although similar to this review, additional papers not identified by the previous reviews have been included as our approach was broader and included all papers reporting on a strategy used to strengthen routine services. For the other reviews, the existence of primary data evaluating the effectiveness or cost-effectiveness of the strategy to improve coverage was required. With this review, primary data on effectiveness of the strategy was not required for inclusion, as we wanted to identify all possible strategies since effectiveness is not always generalizable.

Although routine immunization programs and mass campaigns are complementary strategies used to increase immunization coverage, this review focused on routine immunization programs. This information will be of interest to immunization managers at national and sub-national levels, as well as those interested in increasing population coverage with other recommended health interventions.

## Methods

### Literature Search

We searched on-line library journal databases using 42 terms (Table 1) for papers published in English, French, or Spanish from 1975 through 2004, in total 11,235 papers were identified. We also searched the gray literature by requesting information from 35 websites including WHO regional databases, dissertation, theses and gray literature database websites and contacting 31 experts in the field of which 20 replied and 11 provided references. In total, close to 11,500 papers were identified, the vast majority of which were not routine immunization specific. Based on a review of titles, abstracts and executive summaries, 264 papers were collected. These papers were reviewed by one person and narrowed down to those that presented a study or project conducted to improve routine immunization programs among humans in a low- or middle-income country. Papers assessing immunization campaigns were excluded. Only 60 papers (50 published and 10 unpublished) met these criteria; these papers were then systematically reviewed.

**Table 1:** Search Strategy

**Journal Databases Searched:**	
Ovid MEDLINE, Ovid MEDLINE In-Process & Other Non-Indexed Citations, EMBASE, Sociological Abstracts, CINAHL, ERIC, EBM Reviews – Cochrane Central Register of Controlled Trials, EBM Reviews – Cochrane Database of Systematic Reviews, EBM Reviews – Database of Abstracts of Reviews of Effects, CDSR, ACP Journal Club, DARE, CCTR, Web of Science, CAB Direct, Anthropology Plus, Access UN, Center for Economic Policy Research, Columbia International Affairs Online, GPO Access, CINAHL, Dissertation Abstracts, Hispanic American Periodicals Index, MARCIVE WebDocs, Population Index, World Development Indicators Online, Academic Search Premier, AGRICOLA, ClasePeriodica, EBSCOhost Espanol, Revistas de Investigación	
**Keywords:**	
immunization$, vaccination$, immunization$, Developing Countries, Attitude to Health, Dropouts, Health Service Accessibility, Delivery of Health Care, Community Health Services, Organization and Administration, Primary Heath Care, Comprehensive Health Care, Community Health Centers, Community Health Services, Health Promotion, Health Education, Marketing of Health Services, Health Resources, Communication, Micro planning, Plans of Action, Inter-Agency Coordinating Committee, immunization coverage, vaccination coverage, coverage, immunization uptake, missed opportunities, access, EPI, out reach, supervision, increase coverage, improve coverage, pulse campaign, mobile services, social promotion, social mobilization, reaching every district, immunization plus, universal childhood immunization, UCI, immunization schedule	

**Websites Reviewed:**	**Organizations Contacted:**
	▪ Academy for Educational Development
	▪ Basic Support for Institutionalizing Child Survival
	▪ Centers for Disease Control and Prevention
	▪ Change Project
	▪ Department of Health and Human Services
	▪ International Federation of Red Cross & Red Crescent Societies
	▪ London School of Hygiene and Tropical Medicine
	▪ Program for Appropriate Technology in Health
	▪ Rollins School of Public Health
	▪ Task Force for Child Survival
	▪ United Nations Children's Fund (UNICEF)
	▪ US Agency for International Development
	▪ World Bank
	▪ World Health Organization
	
	
	
	
	
	
	
	
	
	
	
	
	
	
	
	
	
	
	
	
	

### Review Methods

The 60 papers identified through our literature search were first classified into one of three methodological groups: observational studies, studies with evaluation before and after the intervention, and studies with comparison groups. Next, each paper was rated based on a standardized assessment unique to each methodology and data were systematically abstracted. This rating was based on whether elements considered critical to the scientific quality of the study and to the reader's ability to understand adequately the intervention and its impact were present. No effort was made to validate the methods reported, for example that the sample size was correct, rather instead to confirm that the data necessary to verify methods were presented in the paper and that the methods seemed appropriate. The elements assessed included information about and appropriateness of the target population, the use of randomization in the study if appropriate, the presence of clearly defined study questions and outcomes, the identification of possible confounders, the quality of data analysis, evidence of sufficient time to evaluate the intervention, and a discussion of study limitations and how study results compared to published literature (Table 2). The rating ascribed to each paper that described studies with before-and-after interventions or with comparison groups represented a consensus between two reviewers while observational studies were rated by a single reviewer. The same rating was applied to all paper types, however only one reviewer was used for observational studies as many of the criteria we used in the assessment were not applicable for observational studies (e.g. methods for obtaining controls, sample size calculations, accounting for confounders, etc). Papers with a score of more than 60% (number of assessment elements reported in the paper over total elements assessed) were reported and papers with a score below this cut-off were excluded due to difficulty in interpreting the results that they presented. Furthermore papers were excluded that were found to present inconclusive results or focused on strategies to improve the overall health systems as opposed to a particular routine immunization strategy.

**Table 2:** Questions used to assess scientific quality of the study and the reader's ability to adequately understand the intervention

**Questions**	**Trials with Comparison Groups**	**Trials with evaluation before & after**	**Observational**
Is the strategy defined?	Yes	Yes	Yes
Is there a methods section?	Yes	Yes	Yes
Are the strategy and methods sections defined in a comprehensive way?	Yes	Yes	Yes
Is the study question clearly defined?	Yes	Yes	No
Are outcomes/outputs clearly defined?	Yes	Yes	No
Can outcomes be attributed to interventions?	Yes	Yes	No
Is the explanation of the target population sufficient?	Yes	Yes	Yes
Is the target population studied appropriate?	Yes	Yes	No
Was the method used to obtain cases and controls explained?	Yes	No	No
Was randomization used to select cases and controls?	Yes	No	No
Was the method of selecting cases and controls appropriate?	Yes	No	No
Was a method for checking immunization status described?	Yes	Yes	Yes
Was the method for determining sample size provided?	Yes	Yes	No
Have confounders been identified?	Yes	Yes	No
Were the confounders taken into consideration?	Yes	Yes	No
Was the time frame of the study defined?	Yes	Yes	No
Is the time frame sufficient for the intervention to have an impact?	Yes	Yes	Yes
Did the authors make comments regarding limitations of the strategy?	Yes	Yes	Yes
Did the authors compare results with similar studies?	Yes	Yes	Yes
Was adequate data analysis conducted?	Yes	Yes	Yes
Was the response rate adequate?	No	Yes	No

## Results

### Findings

**Figure 1:**
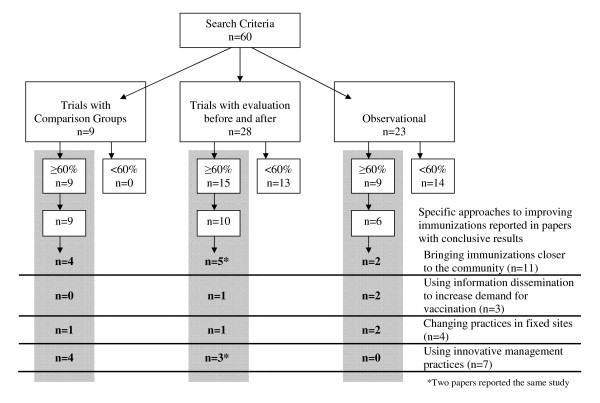
**Review Methods**. see attached file 1.

Only 25 papers met our criteria for inclusion in this review (Table 3). All of the gray literature papers were excluded; most of these papers lacked detailed information or methodology details and so received too low a score to be included. The remaining papers were grouped by the general approach used to improve immunization programs (Figure 1). There were numerous groupings which could have been used to organize the findings, and some papers inevitably overlap. Ultimately, we chose categories that we felt would be most beneficial and the most "user-friendly" for national and sub-national program managers to identify strategies. The four groups were: bringing immunizations closer to communities (n = 11), using information dissemination to increase demand for vaccinations (n = 3), changing practices in fixed sites (n = 4), and using innovative management practices (n = 7). In the selected papers, the most consistently reported outcome indicator was the percentage change in fully vaccinated children (FVC), although some papers used other outcomes such as percentage change in vaccination coverage for specific antigens, dropout from routine immunizations as measured by coverage for an early vaccine when compared to that of a later vaccine, or timeliness of vaccination for a specified antigen.

**Table 3:** Summary of 25 papers reviewed

**Country [Ref] Year(s)**	**Brief Description**	**Study Type**	Change in FVC*
**Bringing immunizations closer to the community**			

Kenya [11] Unknown	Providing outreach immunization services in schools along with dissemination of information about immunizations by students	Trial with evaluation before and after	28% and 32% §
India [6] 1975–1988	Supporting immunization activities in the community by using local women to provide health information and track immunizations	Trial with comparison groups	n/a**
Papua New Guinea [12] 1983–1987	Improving access to immunizations by providing vaccinations at lower level health facilities (health posts) by trained Aid Post Orderlies	Trial with comparison group	n/a
Nigeria [13] 1984–1986	Providing immunizations at more locations and more convenient times in combination with parent education ‡	Trial with evaluation before and after	38%
Mozambique [15,16] 1985–1987	Visiting homes to mobilize the community and refer unvaccinated children to services while providing regular pulse outreach	Trial with evaluation before and after	-4%, 32%, 33% and 14% § ∥
South Africa [8] 1987–1988	Conducting home visits using village health workers who retain visit records ‡	Trial with evaluation before and after	n/a
Bangladesh [7] 1987–1988	Following-up defaulters using low-literacy urban volunteers	Observational	n/a
Ghana [9] 1991–1992	Visiting homes to refer families to services using non-health workers ‡	Trial with comparison groups	19%
Mozambique [14] 1994	Providing outreach services to areas affected by conflict	Observational	n/a
Mexico [10] 1994	Identifying children needing vaccines through home visits by community members	Trial with comparison groups	42%

**Using information dissemination to increase demand for vaccination**			

The West Bank [19] 1985–1996	Developing staffed village-resource rooms	Observational	n/a
Philippines [17] 1989–1990	Communicating measles information through a mass media campaign ‡	Trial with evaluation before and after	11%
Bangladesh [18] 1995	Advocating, by an NGO credit program, for women to utilize immunization services ‡	Observational	n/a

**Changing practices in fixed sites**			

Sudan [21] Unknown	Moving vaccination locations closer to the consulting room or having physicians give an immunization "prescription" after curative care	Observational	n/a
Nigeria [22] 1982	Reorganizing health centers to include a quick immunization line	Trial with evaluation before and after	18%
Mexico [23] 1991	Screening hospitalized children for vaccination status and immunizing those not up-to-date	Observational	n/a
Ethiopia [20] 1991–1992	Using reminder stickers to reduce dropout in fixed facilities along with health education ‡	Trial with comparison groups	n/a

**Using innovative management practices**			

Papua New Guinea [26] 1982–1984	Creating a reporting system based on updated catchment area and target population data, including regular feedback	Trial with evaluation before and after	n/a
Nicaragua [29,30] 1985	Providing food incentives to improve attendance at well child clinics (mobile and fixed) ‡	Trial with evaluation before and after	n/a
Bolivia [25] 1992–1994	Using data and community information to develop appropriate programs	Trial with comparison groups	70%
Indonesia [27] 1993–1994	Training nurses in under-performing health centers using low-cost on-the-job peer training ‡	Trial with comparison groups	n/a
Cambodia [24] 1997–2000	Using contractors to increase immunization coverage and equity ‡	Trial with comparison groups	13% and 1%¶
Madagascar [28] 2000	Using auto-disable syringes for increasing safety and reducing missed opportunities	Trial with comparison groups	n/a

* The change in fully vaccinated children (FVC) may not be comparable across papers as duration of intervention, baseline coverage, and populations vary. For trials with comparison groups, the term "change" represents the difference between groups, whereas for trials with before-and-after evaluations this term represents the change over time.

** n/a indicates that the change in FVC is not reported in the paper.

‡ Paper reported a statistically significant change for vaccination results (α < 0.05).

§ Results for areas reported separately.

∥ Change in FVC before and after intervention in multiple areas.

¶Two different contracting methods were evaluated.

### Bringing immunizations closer to the community

The studies included in this category used non-health workers to encourage people to seek immunization services, or increased access to immunization services by bringing services to communities, and additionally in some cases by increasing demand through educating communities.

Many of these papers documented how the involvement of community members can improve immunization utilization. For example, strengthening demand for immunization services was part of the Integrated Child Development Services Program in India which began in 1975. In this program one village woman for every 1000 population was selected to provide health information to village residents, maintain lists of women and children who needed immunizations, motivate families to bring children for immunizations, assist with immunizations, and follow-up on immunization side effects, as well as to provide other community services. After more than five years of implementation, the proportion of vaccinated children was higher in the intervention group than in the control group for every antigen, ranging from a 35% difference for DPT3 vaccine to a 43% difference for Bacille Calmette-Guerin (BCG) [6].

In Bangladesh and South Africa, tools were developed to assist community workers in tracking their home visits. An observational study in Bangladesh evaluated using semi-literate and illiterate local women in an urban setting to track defaulters using a color-coded tracking system, to refer them to services and accompany mothers to immunization clinics. During the 13-month intervention (1987–1988), 87% percent of children referred by these volunteers completed the recommended immunization series and 96% of women that were referred received tetanus vaccine [7]. A similar program in South Africa evaluated giving record cards to Village Health Workers (VHWs) to record home visits over a one-year period (1988) in an intervention district. VHWs used the cards to identify children to visit, document visit frequency, and track health interventions including immunizations. Sixty-seven percent of children born during the program had completed their third dose of Oral Polio Vaccine (OPV) by eight months of age compared with 50% in the cohort of children (13 to 24 months) born before the program was implemented. However, coverage with measles vaccine by 10 months of age among children aged 13 to 24 months was higher compared to children exposed to the program [8].

In two papers the use of home visits for education and/or service delivery was evaluated. In Ghana, non-health workers conducted door-to-door visits and referred all children less than five years of age to routine immunization clinics. In addition, a health worker conducted home visits for children who failed to finish their immunization series. Over a six-month period (7/1991–2/1992), the percentage of FVC increased from 60% to 85% in the intervention group, whereas in the control group coverage increased from 61% to 67% [9]. In Mexico, trained community members were used to conduct home visits during which immunization education was provided along with needed vaccines. This intervention increased the percentage of FVC less than one year of age from 21% to 77% in five months (1994), compared with the control group where coverage increased only from 30% to 35% [10].

Other successful strategies focused on increasing access to immunization services. In Kenya, school buildings were utilized as immunization centers, with an educational component provided by schoolchildren who circulated immunization information within their communities. Furthermore, mobile teams were used to increase access. Coverage outcomes varied according to population density. In high population density areas the percentage of FVC increased from 54% to 82% and in low density areas it increased from 25% to 57% over an unspecified period. Coverage at follow-up in comparison high density areas was 69% compared to the 82% and in low population density areas 27% compared to the 57% [11]. In a district of Papua New Guinea, health post staff were trained in administering immunizations to permit vaccines to be given closer to rural communities. In this study, conducted between 1983 and 1987, measles coverage increased from 4% to 75% in the intervention district, compared with the control district where coverage increased from 5% to 58% [12]. In Nigeria, access to immunization services was improved by increasing the number of locations offering immunizations and adding mobile clinics in the evenings. The area in which this intervention was conducted saw an increase in FVC from 5% to 43% over a two-year period (1984–1986) [13].

Conflict areas are generally difficult to reach because of security concerns. Three papers evaluated strategies that provided immunizations in conflict areas of Mozambique. Strategies involved using bush planes to gain access to people, providing incentives to attract people to immunization sites, going house-to-house to motivate parents to bring their children for immunization, and working with communities to coordinate provision of services [14-16].

### Using information dissemination to increase demand for vaccination

Information can be provided through numerous channels to either increase awareness of the benefits of immunization or to promote participation. These strategies increase demand for vaccination without changing the service delivery. Mass communication campaigns have the potential to reach large numbers of people, if access to the type of media selected is good. In the Philippines, a mass media campaign focusing on measles vaccination delivered through routine services was evaluated. An increase in the percentage of FVC from 54% in 1989 to 65% in 1990 was reported; this increase was attributed to the impact of the media campaign [17]. In Bangladesh, an increase in immunization coverage was linked to the use of inter-personal communication among mothers participating in a non-government organization (NGO) credit program that encouraged child immunization without providing additional immunization services. Increased coverage of several antigens was reported among the children of women who participated in the NGO program relative to the children of women who did not participate in the NGO program. For example, in 1995, measles coverage was 68% among children of participants compared with 59% in children of non-participants [18]. In the West Bank providing information at the local level through training community members regarding immunizations and providing resource rooms with information on immunization did not increase vaccine coverage, however the timeliness of immunizations, defined as children receiving vaccines at the appropriate age, improved (1985–1996) [19].

### Changing practices in fixed sites

Improved quality of health facility practices can increase coverage through reducing dropout (children that start the vaccination series, but did not complete the series) and missed opportunities (children that were available for vaccination, but that were not vaccinated). In Ethiopia, the use of reminder stickers for parents resulted in decreasing dropout between DPT1 and DPT2 to seven percent in the intervention district compared with 13% in the comparison district during 1992 [20]. A study conducted in Sudan compared two methods to reduce missed opportunities for vaccination: moving the immunization location close to the consultation room in the health facility to provide immediate immunizations to children who had recently been seen in consultation, and having the physician write a prescription for immunizations during curative visits. Each method resulted in an increase of 32% more children being vaccinated during the intervention week than during the week prior to the intervention [21]. An urban Nigerian health center increased coverage of children fully vaccinated by one year of age by 18% in 1982 through reducing wait times by creating a quick immunization line [22]. In a Mexican children's hospital the missed opportunities for immunization were reduced by immunizing all hospitalized children who were not up-to-date with their vaccines. This led to the number of childhood immunizations delivered monthly increasing from 150 to 600 in 1991 [23].

### Using innovative management practices

Reviewed papers addressed two management issues: who should manage immunization systems and how systems might be improved to provide the highest quality services. In Cambodia, increased coverage and improved equity were achieved by contracting immunization services to NGOs in selected districts (1997–2000). Although these districts had higher immunization coverage and improved equity compared to immunization programs run by the Ministry of Health, the annual per capita cost of contracting out services was almost twice that of providing services through the Ministry of Health [24].

Coverage can also be increased through better use of data and community information. In Bolivia, high-risk populations in selected communities were visited biannually from 1992 to 1994 and members of these populations assisted in identifying their priority health problems. Among these targeted populations, 78% percent of children aged 12 to 23 months were fully vaccinated in established programs compared with only eight percent in comparable populations in control communities [25]. Similarly, in Papua New Guinea health staff met to determine how best to improve services. They redefined health facility catchment areas and built a reporting system to collect accurate and meaningful data. These interventions were associated with an increased coverage of DPT2 from 64% in 1980 to 89% in 1984 [26].

Other management methods have been used to improve service quality. In Indonesia, experienced nurses in well-performing health centers peer-trained nurses in poorly-performing health centers (1993–1994). This intervention was low-cost and was associated with increases in coverage for all antigens, such as an increase of 25 percentage points in measles coverage in health centers participating in this program as compared to health centers that did not participate [27]. In Madagascar, in 2000, the use of auto-disable syringes was found to improve the availability of immunizations. Health workers were more willing to vaccinate on non-immunization days since the additional work of syringe and needle sterilization was not required. Missed opportunities for vaccination were thus reduced and coverage was increased. Use of auto-disable syringes also improved injection safety [28]. In Nicaragua, food incentives were introduced (1985) to create demand for immunization services. Mobile outreach without food incentives had 63% attendance but when food incentives were added, attendance increased to 102%. The coverage >100% was described as most likely occurring because of census errors, as mechanisms were put in place to reduce the opportunity for ineligible children to receive the food incentives. A static clinic achieved 94% attendance with food incentives [29,30].

## Discussion

A striking finding from this literature review was the paucity of well-conducted studies examining ways in which routine immunization programs in developing countries may be improved through interventions at the community or facility level. Despite an exhaustive literature search through which we identified greater than 11,000 papers, only 25 were ultimately eligible for inclusion in this review, of which only four projects were conducted in the last ten years. Furthermore, many of these 25 papers were of only moderate scientific quality. This may be in part because scientific research was not the primary purpose of the activity that many of the papers reported. Nonetheless, this situation is surprising in light of the fact that the Expanded Program on Immunization (EPI) has existed for more than a quarter of a century, and the importance and cost-effectiveness of achieving high population coverage with vaccines has been repeatedly recognized [31].

Although every paper included in this review aimed to show an improvement in immunization coverage, a wide range of indicators were used to measure success. A meta-analysis could not be conducted due to the variety of indicators reported. Some strategies were implemented in areas where baseline coverage was relatively high, thus limiting the potential increase in coverage. Other strategies were evaluated in places with low baseline coverage, and thus had the potential to result in large coverage increases. For these reasons, it is difficult to determine which strategies were most successful. Furthermore, some strategies may be more successful in certain social or health care settings than others. It is challenging to determine the generalizability of the findings, as less than half of the papers included a complete discussion of findings including topics such as comparison of findings from other similar studies.

The 25 papers identified reveal how community and facility-based strategies to strengthen routine immunization programs may result not only in increased vaccination coverage, but in other benefits. For example, projects designed to increase coverage were associated with improved timeliness of vaccination, improved knowledge regarding vaccines, improved quality, and increased equity.

Evidence from the papers suggests non-health workers can provide numerous services including education, mobilization, and tracking of target populations. Often these non-health workers are very successful because of their community knowledge, the respect they are given by the community, and the fact that they have access to community members who may not be reached by mass media such as radio or television. Community members can be used to promote specific antigens based on their expertise; for example, Traditional Birth Attendants (TBAs) may be best at increasing coverage of vaccines delivered early in life (i.e., BCG, DPT1). Home visits by non-health – worker volunteers can be very successful at motivating parents to utilize immunization services. During house visits, these volunteers can identify families not utilizing services; these families can then be followed-up by health workers.

In general, the sustainability of interventions as perceived by papers' authors was not addressed in the papers reviewed, and few programs were evaluated for enough time to determine sustainability. Given the number of interventions relying on volunteers, sustainability is of particular concern. Researchers should evaluate the residual impact of the intervention, to better understand the sustainability of the project. For example evaluating if the program resulted in change in infrastructure or practices that would continue to improve immunization coverage after the project was over. In this paper, no data have been presented on the cost-effectiveness of the various interventions discussed. These data have not been included since the two other reviews, previously mentioned [4,5], have reviewed and published the cost-effectiveness, as well as effectiveness, of various immunization service strategies.

Our literature review had a number of limitations. Although we attempted to conduct a thorough search for papers, those not readily available through databases, or on the web may have been missed. Furthermore the literature-gathering process was conducted through the use of a computer from an office; a more complete review may have been achieved through visiting locations to access literature in person [5]. The methods used to assess the quality of papers and thus determine their eligibility for inclusion in this review may have been biased toward published papers as many gray literature papers did not discuss the study methodology used in enough detail to allow it to be assessed. As such, no gray literature papers were included in this review. Furthermore, the majority of papers, published and unpublished, reported positive results, thus excluding opportunities to learn from unsuccessful interventions.

### Additional Research

Although a wide range community and facility-based strategies to strengthen immunization programs were covered in the papers reviewed, there remain many areas for further research. Some of the topics we felt were lacking from the papers reviewed may not be specific to immunization and as such were not identified through our search strategy. For example, we found few studies related to health facility management, facility staffing, or community financing of health facilities. However, it seems likely that such studies exist. Topic areas which we identified as important and were likely not missed due to our search strategy were highlighted as needing further research (Table 4). Attention to these areas will be important as immunization services are integrated with other health interventions and new vaccines are introduced. Studies should strive to use rigorous scientific methods, for example by calculating minimum sample sizes based on clearly articulated assumptions, assessing confounders, using control areas when appropriate, using randomization to select intervention areas, and using statistical tests as indicated in data analysis. Furthermore, the results of these studies should be widely disseminated. In addition to peer-reviewed publication, studies can be disseminated through Interagency Coordinating Committees, newsletters, WHO regional bulletins, and press releases.

**Table 4:** Proposed Areas of Additional Research

1. Integration and collaboration
▪ How feasible and cost-effective is it to integrate other services with routine immunizations?
▪ In what circumstances should integrated programs be considered?
▪ What are optimal services or packages of services to integrate with routine immunizations?
▪ Can an increased role for private providers and non-governmental organizations strengthen routine immunization services?
▪ Can additional groups (i.e. local service groups) be used to promote routine immunizations by providing positive immunization messages and long-term communication?
▪ Can increased involvement of civil society organizations at each level also improve accountability, service delivery and coverage?
▪ How best to work with partners to improve overall service delivery and thus strengthen routine immunization services?
2. New Vaccine Introduction
▪ What are the barriers to the introduction of new vaccines at community and facility level, and how can these be overcome?
3. Service Delivery
▪ What are the benefits of supportive supervision?
▪ How can a supportive supervision environment be created?
▪ What are the best roles for community volunteers?
▪ What are potential roles for existing community and leadership structures (not just volunteers)?
▪ What are predictors of sustainability for volunteer-based programs?

## Conclusion

This paper summarizes the literature in terms of what is reported to have been successful in improving routine immunization programs through community and facility-based interventions over the past thirty years. Information obtained from well conducted scientific studies will be crucial to assist program managers to implement strategies to achieve high coverage. These activities coupled with the attention being given to the health systems approached advocated by the GAVI Alliance and other donors will be crucial to ensure that all levels of the immunization system function effectively. The potential health improvements from vaccines will continue to increase as new vaccines become available and as the price of these vaccines becomes more affordable. Nonetheless, as the true impact of vaccination depends heavily on the ability of immunization programs to reach every targeted individual without clear local delivery strategies countries will not be in a position to take full advantage of the potential for reduction of disease. Data from these papers confirms the need for well managed immunization programs providing high quality accessible services in conjunction with community demand. These key elements are also the basis of the WHO Reaching Every District (RED) strategy [32], a broad, all-encompassing approach covering five areas of immunization programs. Findings from this paper also indicate that all elements of an immunization program need to be addressed. For example, easily-accessed, high-quality services will not be utilized if community demand is lacking. With this in mind, it is critical to build upon lessons from the past and to continue to conduct research on how high vaccination coverage can be achieved in every community.

## Competing interests

The authors declare that they have no competing interests.

## Authors' contributions

TKR: Made substantial contributions to conception and design, acquisition of data, analysis and interpretation of data. Has also been involved in drafting the manuscript, revised it critically for important intellectual content and given final approval for the version to be published. VD: Made substantial contributions to conception and design, acquisition of data, analysis and interpretation of data. Has also been involved in drafting the manuscript, revised it critically for important intellectual content and given final approval for the version to be published. KLC: Has been involved in drafting the manuscript, revised it critically for important intellectual content and given final approval for the version to be published.

## Pre-publication history

The pre-publication history for this paper can be accessed here:



## References

[B1] World Health Organization [Internet] Immunization surveillance, assessment and monitoring: vaccine preventable diseases. http://www.who.int/immunization_monitoring/diseases/en/.

[B2] World Health Organization [Internet] Immunization surveillance, assessment and monitoring: vaccine preventable diseases. http://www.who.int/immunization_monitoring/data/SlidesGlobalImmunization.pdf.

[B3] Infectious Diseases in Children [Internet] Vaccines for Children in the Pipeline. http://www.idinchildren.com/200607/vaccines.pdf.

[B4] Pegurri E, Fox-Rushby JA, Walker D (2005). Effects, costs and cost-effectiveness of interventions to expand coverage of immunisation services in developing countries: A systematic review of the published literature. Vaccine.

[B5] Batt K, Fox-Rushby J, Castillo-Riquelme M (2004). The costs, effects and cost-effectiveness of strategies to increase coverage of routine immunizations in low- and middle- income countries: systematic review of the grey literature. Bulletin of the World Health Organization.

[B6] Tandon BN, Gandhi N, the Integrated Child Development Consultants (1992). Immunization coverage in India for areas served by the Integrated Child-Development Services programme. Bulletin of the World Health Organization.

[B7] Hughart N, Silimperi DR, Khatun J, Stanton B (1992). A new EPI strategy to reach high risk urban children in Bangladesh: urban volunteers. Trop Geogr Med.

[B8] Kuhn L, Zwarenstein M (1990). Evaluation of a village health worker programme: the use of village health worker retained records. International Journal of Epidemiology.

[B9] Brugha RF, Kevany JP (1996). Maximizing immunization coverage through home visits: a controlled trial in an urban area of Ghana. Bulletin of the World Health Organization.

[B10] Calderón-Ortiz R, Mejía-Mejía J (1996). Estrategia de contratación permanente dentro del Programa de Vacunación Universal. Salud Pública de México.

[B11] Anonymous (1977). Expanded Programme on Immunization: Study of feasibility, coverage and cost of maintenance immunization for children by district mobile teams in Kenya. Weekly Epidemiological Record.

[B12] Alto WA, Alk S, Pinau D, Polume H (1989). Improving immunization coverage, a comparison between traditional MCH teams and MCH teams plus Aid Post Orderlies. P N G Med J.

[B13] Oruamabo RS, Okoji GO (1987). Immunisation status of children in Port Harcourt before and after commencing the Expanded Programme on Immunisation. Public Health.

[B14] Coninx R, Dupuy C, Hermann C, Ribeiro GC, Margot M, Lucic K (1998). Vaccination of the civilian population in a country at war: it can be done; it can also be evaluated. The ICRC experience in Mozambique Journal of Tropical Pediatrics.

[B15] Cutts FT, Kortbeek S, Malalane R, Penicele P, Gingell K (1988). Developing appropriate strategies for EPI: A case study from Mozambique. Health Policy & Planning.

[B16] Cutts FT, Phillips M, Kortbeek S, Soares A (1990). Door-to-door canvassing for immunization program acceleration in Mozambique: Achievements and costs. International Journal of Health Services.

[B17] Zimicki S, Hornik RC, Verzosa CC, Hernandez JR, deGuzman E, Dayrit M, Fausto A, Lee MB, Abad M (1994). Improving vaccination coverage in urban areas through a health communication campaign: the 1990 Philippine experience. Bulletin of the World Health Organization.

[B18] Amin R, Li YP (1997). NGO-promoted women's credit program, immunization coverage, and child mortality in rural Bangladesh. Women & Health.

[B19] Tulchinsky T, Al Zeer AM, Abu Mounshar J, Subeih T, Schoenbaum M, Roth M, Gamulka B, Abenueze M, Acker C (1997). A successful, preventive-oriented village health worker program in Hebron, the West Bank, 1985–1996. J Public Health Manag Pract.

[B20] Berhane Y, Pickering J (1993). Are reminder stickers effective in reducing immunization dropout rates in Addis Ababa, Ethiopia?. J Trop Med Hyg.

[B21] Loevinsohn BP, Gareaballah E (1992). Missed opportunities for immunization during visits for curative care: a randomized cross-over trial in Sudan. Bulletin of the World Health Organization.

[B22] Ekunwe EO (1984). Expanding immunization coverage through improved clinic procedures. World Health Forum.

[B23] Avila-Figueroa C, Navarrete-Navarro S, Ramírez-Galván L, Baltazar-López A, López-Serrano M, Santos-Preciado JI (1992). Inmunizaciones en niños hospitalizados y de consulta externa: reducción de las oportunidades perdidas de vacunación. Bol Med Hospital Infant Mex.

[B24] Schwartz JB, Bhushan I (2004). Improving immunization equity through a public-private partnership in Cambodia. Bulletin of the World Health Organization.

[B25] Perry H, Robison N, Chavez D, Taja O, Hilari C, Shanklin D, Wyon J (1998). The census-based, impact-oriented approach: its effectiveness in promoting child health in Bolivia. Health Policy and Planning.

[B26] van Zwanenberg TD, Hull C (1988). Improving immunisation: coverage in a province in Papua New Guinea. Br Med J (Clin Res Ed).

[B27] Robinson JS, Burkhalter BR, Rasmussen B, Sugiono R (2001). Low-cost on-the-job peer training improved immunization coverage in Indonesia. Bulletin of the World Health Organization.

[B28] Drain PK, Ralaivao JS, Rakotonandrasana A, Carnell MA (2003). Introducing auto-disable syringes to the national immunization programme in Madagascar. Bulletin of the World Health Organization.

[B29] Loevinsohn BP, Loevinsohn ME (1986). Improvement in coverage of primary health care in a developing country through use of food incentives. The Lancet.

[B30] Loevinsohn BP, Loevinsohn ME (1987). Well child clinics and mass vaccination campaigns: an evaluation of strategies for improving the coverage of primary health care in a developing country. American Journal of Public Health.

[B31] Hadler SC, Cochi SL, Bilous J, Cutts FT, Plotkin SA, Orenstein WA (2004). Chapter 55: Vaccination Programs in Developing Countries. Vaccines.

[B32] World Health Organization [Internet] The RED Strategy. http://www.who.int/immunization_delivery/systems_policy/red/en/.

